# Impact of wearable wireless continuous vital sign monitoring in abdominal surgical patients: before–after study

**DOI:** 10.1093/bjsopen/zrad128

**Published:** 2024-01-17

**Authors:** Jobbe P L Leenen, Vera Ardesch, Cor J Kalkman, Lisette Schoonhoven, Gijs A Patijn

**Affiliations:** Department of Surgery, Isala, Zwolle, The Netherlands; Connected Care Centre, Isala, Zwolle, The Netherlands; Research Group IT Innovations in Healthcare, Windesheim University of Applied Sciences, Zwolle, The Netherlands; Flex pool department, Isala, Zwolle, The Netherlands; Department of Anaesthesiology, University Medical Centre Utrecht, Utrecht University, Utrecht, The Netherlands; Julius Centre for Health Sciences and Primary Care, University Medical Centre Utrecht, Utrecht University, Utrecht, The Netherlands; School of Health Sciences, Faculty of Environmental and Life Sciences, University of Southampton, Southampton, UK; Department of Surgery, Isala, Zwolle, The Netherlands; Connected Care Centre, Isala, Zwolle, The Netherlands

## Abstract

**Background:**

Technological advances have enabled continuous monitoring of vital signs (CMVS) by wearable, wireless devices on general hospital wards to facilitate early detection of clinical deterioration, which could potentially improve clinical outcomes. However, evidence on the impact of these CMVS systems on patient outcomes is limited. This research aimed to explore the effect of CMVS on the clinical outcomes in major abdominal surgery patients in a general surgery ward.

**Methods:**

A single-centre before–after study was conducted from October 2019 to June 2022. Patients in the intervention group received CMVS in addition to conventional intermittent vital sign monitoring (standard care for control group). With CMVS, heart rate and respiratory rate were measured every 5 min by a patch sensor. Proactive vital signs trends assessments and, when necessary, subsequent nursing activities were performed every nursing shift. The primary outcome of interest was the length of hospital stay (LOS); also, 12 patient-related outcomes were analysed. In the CMVS group, follow-up nursing activities of deviating vital signs trends were described and patient acceptability was measured. Post-hoc subgroup analysis was performed for colorectal and hepatopancreatobiliary surgery.

**Results:**

A total of 908 patients were included (colorectal: *n* = 650; hepatopancreatobiliary: *n* = 257). Overall, median LOS was lower in the CMVS group (5.0 *versus* 5.5 days; *P* = 0.012), respectively. Post-hoc subgroup analysis showed this reduction in LOS was mostly observed in the colorectal group and not in the hepatopancreatobiliary group. Apart from a decrease in nurse-to-house-officer calls (from 15.3% to 7.7%; *P* = 0.007), all secondary clinical outcomes were similar in CMVS and control groups. However, a non-significant trend towards less-severe complications and reduced ICU LOS was observed in the CMVS group. In CMVS patients, 109 additional nursing activities were performed and 83% of patients indicated CMVS was acceptable.

**Conclusion:**

CMVS was associated with a significant reduction in LOS, while other clinical outcomes were unchanged. CMVS triggered additional nursing activities such as extra patient assessments and therapeutic interventions.

## Teaser

A before–after study including 908 abdominal surgery patients showed wearable continuous vital signs monitoring on the general ward was associated with a significant decrease in length of stay. Besides a decrease in nurse-to-house-officer calls, secondary outcomes were similar in intervention and control groups. Also, the intervention triggered additional interventions such as extra patient assessments and therapeutic interventions.

## Introduction

Postoperative complications after major abdominal surgery may occur in up to 44% of all patients^1,2^, impact a broad range of patient outcomes and also considerably increase costs^3–10^. They not only increase mortality and prolong hospital stay, but also result in the need for an increased level of post-discharge care and a higher readmission rate. Furthermore, long-term outcomes such as quality of life and functional performance are negatively affected^10,11^.

Severe postoperative complications are commonly associated with clinical deterioration and, when detected early, timely intervention may reduce morbidity and mortality^12^. Vital sign deviations usually precede clinical deterioration. To promote identification of patients at risk, simple physiological parameter-based protocols are broadly implemented on general wards^13,14^. Generally, the five key vital signs^15^ (blood pressure, blood oxygen saturation, heart rate, respiratory rate and body temperature) are measured manually 1–3 times a day in general wards and aggregated into a single number using the Early Warning Scores (EWS) system. A critical limitation of these systems is that the physiological measurements are intermittent, there is poor protocol adherence and sometimes inaccurate vital sign recording^16–18^. Patients may unexpectedly deteriorate, which may go unnoticed in between routine vital signs measurements^19^.

Over the last decade, new technological advances facilitated the introduction of continuous monitoring of vital signs (CMVS) by wearable, wireless devices on general wards. These CMVS interventions allow earlier detection of clinical deterioration and may improve clinical outcomes, in particular reduced complication severity, reduction of failure to rescue events and fewer ICU admissions, all of which combined may decrease total length of stay^12,18,20–22^. However, evidence for a positive effect on clinical outcomes in general ward patients with wearable devices is scarce^23,24^. This may be explained by the challenging implementation of CMVS in clinical workflows^25–27^.

Successful implementation is essential before any potential effectiveness of continuous monitoring can be reliably demonstrated. Therefore, a CMVS intervention was developed and its feasibility evaluated in two previous studies^28,29^. Subsequently, an interventional study with a hybrid design focusing on both evaluation of the implementation and the effectiveness of the intervention was set up^30^. The success of this implementation strategy is described elsewhere^31^. Here, the findings regarding the impact of CMVS on the surgical ward on clinical outcomes in major abdominal surgery patients, consisting of elective colorectal and hepatopancreatobiliary (HPB) surgery, are described as compared to a historical control group. The primary aim was to explore the effect of CMVS on length of hospital stay (LOS). Secondary aims were to explore the effects of CMVS on a broad range of other clinical outcome measures.

## Methods

### Study design

A single-centre before–after study as part of a type 2 hybrid design^30^ was conducted from October 2019 to July 2022 in a 1250-bed teaching hospital in the Netherlands. This study is reported in concordance with the STROBE guidelines and was registered in the ISRCTN registry (ISRCTN37125996)^32^.

### Participants and setting

Patients admitted to the surgical ward for elective major abdominal surgery, including both colorectal (average of 230 surgeries per year) and HPB (average of 115 surgeries per year) resections, were eligible to participate in the study. Inclusion criteria were: ≥ 18 years old and expected hospitalization of ≥2 days. Patients admitted between October 2019 and November 2021 were retrospectively included in the pre-implementation group as controls. From November 2021 to June 2022, patients were prospectively included in the intervention group (post-implementation group). No substantive changes were made to unit staffing, or to hospital protocols, departmental safety and quality policies during the 2.5-year study period. Patients were excluded when the primary indication for hospitalization was acute (not elective), had a palliative indication, a known allergy for any of the materials of the sensor or when they participated in another (potentially conflicting) study.

### Continuous monitoring of vital signs intervention

Pre-implementation, the standard of care was intermittent manual monitoring using the Modified Early Warning Score (MEWS) every 24 h according to the local hospital protocol. For every MEWS, besides subjective measurements, five vital signs were recorded: respiratory rate (RR), heart rate (HR), blood pressure (BP), core temperature and oxygen saturation^33^ (*Supplementary material*). Vital signs were measured manually using a blood pressure measuring device with a pulse oximeter, an ear thermometer, and by visual inspection of RR.

Post-implementation, in addition to the standard MEWS protocol, patients were continuously monitored by the Conformité Européene marked Philips Healthdot and Intellivue Guardian Solution (IGS) software system (Philips, Eindhoven, The Netherlands). The wireless monitoring sensor is embedded in a patch worn on the patient’s chest (*Supplementary Material*, *Fig. S1*); it continuously records heart rate (HR) in beats per minute and respiratory rate (RR) in respirations per minute using a chest accelerometer. Every 5-min interval, the vital signs measurements were wirelessly transmitted to the IGS software. Within the IGS software, vital sign trends are visualized and, complementary to the hospital MEWS protocol, a partial MEWS-score (D-EWS) was aggregated every hour based on the thresholds for HR and RR. As the device measures only two vital signs, the intervention was used in addition to the standard-of-care intermittent manual measurements. Based on the feasibility study findings, instead of using an alarm strategy, nurses routinely assessed current vital signs and their trends every 4 h (that is, twice per 8-h shift) without alarms^29^. At the end of every shift, they reported the D-EWS score, possible abnormalities, deviations and subsequent nursing activities in the electronic health record (EHR). A full description of the intervention and implementation strategy is reported elsewhere^34^.

### Outcomes of interest

The primary outcome for the study was the effect of CMVS in hospital LOS in days. Discharge time before 2p.m. was considered as a 0.5-day based on routine workflows for operating room and ward bed capacity. Secondary postoperative outcomes were divided into in-hospital and post-discharge outcomes. In-hospital outcomes were: proportion of long LOS (defined as +1 standard deviation or third quartile or higher of the control group); rapid response team (RRT) calls; nurse-to-house-officer (HO) calls (defined as junior resident calls between 6 p.m. and 8 a.m.) regarding deviating vital signs; unplanned ICU admissions, ICU LOS, reoperations; mortality <30 days after surgery; severe complications (severity IIIa to V according to the Clavien–Dindo classification^38^); and postoperative unplanned CT or MRI scans. Post-discharge outcomes were: readmissions <30 days after discharge; days alive at home^39^ (DAH_30_); discharge disposition; and type and amount of required post-discharge nursing care.

All nursing activities that were initiated and performed based on the CMVS trend assessments were documented by the nurses. These were divided into performing additional checks or interventions in consultation with a physician. In addition, patients completed a questionnaire consisting of the acceptability intervention measurement (AIM), a patient-reported experience measurement about comfort of the sensor and recommendation score on a scale of 1–5, an overall score on a scale of 1–10 and free space for remarks. The AIM questionnaire consisted of four statements about acceptance on a 5-point Likert scale (score 1–5). A median score of ≥ 3.5 was defined as sufficient acceptability^40^.

The following patient characteristics were collected: gender, age, length, weight, BMA, ASA classification, procedure (laparoscopic or open), malignancy (none, solid tumour or metastasis), nutritional status (the short nutritional assessment questionnaire score with score ≥3 or higher as malnutrition^35^), smoking status (yes, no or prior), alcohol use (yes, no), preoperative haemoglobin (Hb), co-morbidities (Charlson Comorbidity Index (CCI) score ranging 0–12) and MEWS measurement frequency^36,37^. For the CMVS group, the distribution of D-EWS scores was presented.

### Study size

Estimation of the sample size was calculated with MedCalc (MedCalc Software Ltd, Ostend, Belgium). A two-tailed alpha of 5%, power of 0.80 and LOS (in hours) with 150.1 (intervention) *versus* 187.7 h (control) in ratio 1:4, resulted in at least 180 patients required for the intervention group and 720 for the control group. LOS in the CMVS group was prospectively recorded, and LOS in the control group was derived from the hospital EHR. An additional 10% for potentially non-parametric testing resulted in at least 198 patients in the intervention group and 792 patients in the control group.

### Statistical analysis

LOS was compared between the CMVS and control groups. Multiple imputation was performed to handle missing data when present. Normally distributed continuous data were presented as means and s.d. and tested with unpaired *t*-tests. Likewise, non-normally distributed data are presented as median and i.q.r. and were tested with Mann–Whitney U-tests. Normality was checked by the Kolmogorov–Smirnov test and visually by a Q-Q plot and histogram. Nominal data were presented with frequencies and percentages (*n*, %) and tested with χ² test or Fisher exact tests based on assumptions. Post-hoc analysis of the subgroups colorectal and HPB surgery were performed to compare outcomes between CMVS and control groups.

A multivariable analysis to determine impact of CMVS on log-transformed LOS was performed while controlling for gender, type of surgery (colorectal or HPB), procedure, Charlson Comorbidity Index, significant different co-morbidities, complications and group. Multicollinearity was present if the variance inflation factor was ≥5. Over time, effects were analysed by comparison of median LOS over years. All data were analysed with IBM SPSS Statistics 26 for Windows (IBM Armork, New York, USA) and *P* < 0.05 was considered significant.

### Ethics

The Daily Board of the Medical Ethics Committee Isala reviewed the protocol (protocol 20211114) and declared the Medical Research Involving Human Subjects Act (also known by its Dutch abbreviation ‘WMO’) did not apply for the study. The study was conducted in accordance with the Declaration of Helsinki. Written informed consent was obtained from patients participating in the post-implementation group. A waiver was provided for patients in the pre-implementation group.

## Results

### Study characteristics

**Fig. 1:**
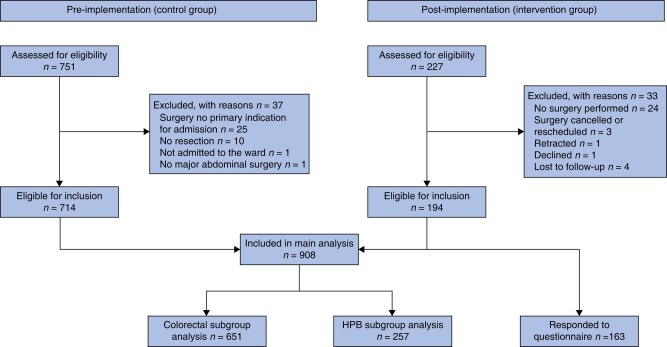
Flowchart of the study HPB, hepatopancreatobiliary.

A total of 978 patients were screened and after exclusion, 908 were eligible for analysis: 714 controls and 194 intervention patients (*Fig. 1*). Proportion of ASA class 3–4 was higher in the CMVS group (35.1% *versus* 25.9%; *P* = 0.012) although the CCI score was lower for the CMVS group (5.2 *versus* 5.8; *P* = 0.004; *Table 1*). *Table 1* presents the characteristics of all patients.

**Table 1:** Patient characteristics

	Control group (*n* = 714)	CMVS group (*n* = 194)	*P*
**Sex**			0.313
Male	361 (50.6)	106 (54.6)	
Female	353 (49.4)	88 (45.4)	
Age, mean (s.d.)	66.8 (13.1)	68.1 (11.9)	0.236
Length, mean (s.d.)	172.8 (11.7)	174.1 (10.2)	0.163
Weight, mean (s.d.)	79.0 (15.9)	80.5 (16.8)	0.258
BMI, mean (s.d.)	26.3 (4.7)	26.5 (4.7)	0.607
**ASA classification**			0.012*
1–2	529 (74.1)	126 (64.9)	
3–4	185 (25.9)	68 (35.1)	
**Type**			0.399
Colorectal	507 (71.0)	143 (74.1)	
HPB	207 (29.0)	50 (25.9)	
**Procedure**			0.299
Laparoscopic	552 (73.1)	149 (76.8)	
Open	192 (26.9)	45 (23.3)	
**Malignancy**			0.310
No tumour	126 (17.6)	35 (18.0)	
Solid tumour	480 (67.2)	138 (71.1)	
Metastasis	108 (15.1)	21 (10.9)	
CCI, mean (s.d.)	5.8 (2.8)	5.2 (2.5)	0.004*
Myocardial infarction	67 (9.4)	18	1.000
COPD	83 (11.6)	10 (5.2)	0.011*
Hypertension	360 (50.4)	46 (23.7)	0.000*
Cerebral vascular accident	88 (12.3)	16 (8.2)	0.128
Chronic heart failure	39	4	0.055
Chronic kidney disease	11	0	0.211
Dementia	4	2	0.614
Connective tissue disease	17	3	0.592
Peptic ulcer disease	9	2	1.000
Liver disease	6	0	0.351
Hemiplegia	3	1	1.000
Leukaemia	5	1	1.000
Lymphoma	23	1	0.074
AIDS	0	0	n/a
Preoperative Hb, mean (s.d.)	7.9 (1.1)	7.7 (1.2)	0.061
**Smoking status**			0.963
No	281 (39.4)	75 (38.7)	
Prior	353 (49.4)	96 (49.5)	
Yes	80 (11.2)	23 (11.9)	
Alcohol use	364 (51.0)	88 (45.6)	0.184
**Nutritional status**			0.161
No malnourishment	584 (81.8)	167 (86.1)	
Malnourishment	130 (18.2)	27 (13.9)	
MEWS measurement frequency, median (i.q.r.)	1.7 (1.3–4.1)	1.6 (1.2–4.3)	0.217
Hours of CMVS, mean (s.d.)	n/a	118 (105)	n/a
**D-EWS**	n/a	17 176	n/a
Score 0		3853 (22.4)	
Score 1–2		12 670 (73.8)	
Score 3 or higher		653 (3.8)	

Values are *n* (%) unless otherwise stated. HPB, hepatopancreatobiliary; CCI, Charlson Comorbidity Index; CMVS, continuous vital sign monitoring; D-EWS, partial MEWS; Hb, haemoglobin; MEWS, Modified Early Warning Score; n/a, not applicable. *Statistically significant.

In the post-hoc subgroup analysis, several statistically significant baseline differences were present (*Table 2*). In the colorectal CMVS group, less rectal resections were performed and perioperative Hb and ASA classification were higher. In the HPB CMVS-group, more pancreas resections were performed, CCI score was lower, and there were more active smokers in comparison with the control group.

**Table 2:** Patient characteristics of subgroups

	Colorectal surgery (*n* = 651)			HPB surgery (*n* = 257)		
	Controls (*n* = 507)	CMVS (*n* = 144)	*P*	Control (*n* = 207)	CMVS (*n* = 50)	*P*
**Sex**			0.502			0.343
Male	248 (48.9)	75 (52.1)		113 (54.6)	31 (62.0)	
Female	259 (51.1)	69 (47.9)		94 (45.4)	29 (58.0)	
Age, mean (s.d.)	67.0 (13.5)	68.6 (12.2)	0.201	66.3 (12.0)	66.4 (11.0)	0.962
Length, mean (s.d.)	172.7 (9.8)	174.0 (10.3)	0.188	173.0 (15.3)	174.5 (9.8)	0.522
Weight, mean (s.d.)	79.1 (16.1)	80.9 (16.0)	0.238	78.6 (15.6)	79.4 (19.5)	0.828
BMI, mean (s.d.)	26.4 (4.8)	26.6 (4.4)	0.553	26.0 (4.4)	26.0 (5.6)	0.948
**ASA classification**			0.008*			0.449
1–2	393 (77.5)	96 (66.7)		136 (65.7)	30 (60.0)	
3–4	114 (22.5)	48 (33.3)		71 (34.4)	20 (40.0)	
**Type**			0.011*			0.019*
Colon/liver	366 (72.2)	119 (82.6)		100 (51.7)	15 (70.0)	
Rectal/pancreas	141 (27.8)	25 (17.4)		107 (48.3)	35 (30.0)	
**Procedure**			0.288			0.883
Laparoscopic	441 (87.0)	130 (90.3)		81 (39.1)	19 (38.0)	
Open	66 (13.0)	14 (9.7)		126 (60.9)	31 (62.0)	
**Malignancy**			0.563			0.058
No tumour	103 (20.3)	26 (18.1)		23 (11.1)	9 (18.0)	
Solid tumour	382 (75.3)	109 (75.7)		98 (47.3)	29 (58.0)	
Metastasis	22 (4.3)	9 (6.9)		86 (41.5)	12 (24.0)	
CCI, mean (s.d.)	5.4 (2.6)	5.1 (2.3)	0.225	6.8 (3.0)	5.5 (2.9)	0.005*
Preoperative Hb, mean (s.d.)	7.9 (1.1)	7.7 (1.2)	0.038*	8.0 (1.1)	8.0 (1.1)	0.967
**Smoking status**			0.197			0.002*
No	202 (39.8)	64 (44.4)		79 (38.2)	11 (22.0)	
Prior	244 (48.1)	70 (48.6)		109 (52.7)	26 (52.0)	
Yes	61 (12.0)	10 (7.0)		19 (9.2)	13 (26.0)	
Alcohol use	276 (54.4)	70 (486)	0.216	88 (42.5)	19 (38.0)	0.561
**Nutritional status**			0.239			0.581
No malnourishment	439 (86.6)	130 (90.3)		145 (70.0)	37 (74.0)	
Malnourishment	68 (13.4)	14 (9.7)		62 (30.0)	13 (26.0)	

Values are n (%) unless otherwise stated. HPB, hepatopancreatobiliary; CMVS, continuous vital signs monitoring intervention; CCI, Charlson Comorbidity Index; Hb, haemoglobin. *Statistically significant.

### Length of stay

Median (i.q.r.) LOS for the total CMVS group was 5.0 (3.5–8.6) days *versus* 5.5 (4.0–10.0) days in the control group (*P* = 0.012; *Table 3*). After controlling for patient and surgical characteristics with multivariate analysis, this difference was maintained with an unstandardized coefficient of −0.043 (95% c.i. −0.077 to −0.009). Except for gender and CCI score, all other variables in the model added statistically significantly to the prediction of LOS. Significant different co-morbidities such as hypertension and chronic obstructive pulmonary disease were added to the model in a separate analysis but did not change the outcome (*Table S1*).

**Table 3:** Clinical outcomes of major abdominal surgery

	Control group (*n* = 714)	CMVS group (*n* = 194)	*P*
Length of stay, median (i.q.r.)	5.5 (4.0–10.0)	5.0 (3.5–8.6)	0.012‡*
In-hospital outcomes			
Long LOS	179 (25.1)	40 (20.6)	0.199
Rapid response team calls	2	3	0.068†
House Officer calls	109 (15.3)	15 (7.7)	0.007‡
ICU admissions	17	3	0.592†
ICU LOS, median (i.q.r.)	7.0 (3.0–18.5)	3.0 (2.25–3.00)	0.132a
Mortality	5	1	1.000†
Reoperations	53 (7.4)	14 (7.2)	0.922
**Unplanned diagnostics**			
CT	96 (13.4)	28 (14.4)	0.722
MRI	5	0	0.590†
Complication rate	70 (9.8)	19 (9.8)	0.997
**Complication severity**	83 (100.0)	23 (100.0)	0.808†
IIIa	25	7	1.000†
IIIb	41	13	0.545
IVa	8	2	1.000†
IVb	6	0	0.336
V	3	1	1.000†
Post-discharge outcomes			
Readmissions	77 (10.8)	25 (12.9)	0.411
Readmissions’ LOS, median (i.q.r.)	6.5 (4.0–9.5)	4.0 (2.0–7.0)	0.014‡*
DAH_30_, median (i.q.r.)	24.0 (18.9–26.0)	24.5 (20.5–26.5)	0.005‡*
**Discharge disposition**			
Independent	467 (65.4)	116 (59.8)	0.148
Home care	211 (29.6)	75 (38.9)	0.015‡
Rehabilitation centre	24	2	0.093
Nursing home	3	0	1.000†
Other ward	5	0	0.590†
Deceased	3	1	1.000†
Hospice	1	0	1.000†
**Frequency of post-hospital care**			0.123
1	152 (61.8)	44 (61.1)	
2	66 (26.8)	25 (34.7)	
≥ 3	28 (11.4)	3 (4.2)	
**Type of post-hospital care**			
ADL	116 (16.2)	33 (17.1)	0.827
Stoma	114 (16.0)	24 (12.4)	0.259
Medication	77 (10.8)	26 (13.5)	0.307
Wound care	57 (8.0)	19 (9.8)	0.384
Tube feeding	17	6	0.605†
Drain care	14	12	0.005‡
Catheter	13	6	0.263†
Other	6	0	0.351

Values are *n* (%) unless otherwise stated. ADL, activities of daily living; LOS, length of stay; DAH_30_, days alive at home 30 post-surgery. *Mann–Whitney U-test. †Fisher Exact test. ‡Statistically significant.

Post-hoc subgroup analysis showed that in patients undergoing colorectal procedures, LOS in the CMVS group was lower than in the control group (median LOS 4.0 *versus* 4.5 days; *P* = 0.001). In the patients undergoing HPB surgery, median LOS was similar between the CMVS and control groups (9.0 *versus* 9.0 days; *P* = 0.754; *Table 4*). After multivariate analysis, this difference was maintained for the colorectal patients, whereas LOS remained similar in HPB patients (*Table S1*). No time effect was present (*Supplementary Material*, *Table S2*).

**Table 4:** Clinical outcomes of subgroups

	Colorectal surgery (*n* = 651)			HPB surgery (*n* = 257)		
	Control (*n* = 507)	CMVS (*n* = 144)	*P*	Control (*n* = 217)	CMVS (*n* = 50)	*P*
Length of stay, median (i.q.r.)	4.5 (3.5–7.5)	4.0 (3.0–6.0)	0.001‡*	9.0 (6.0–15.0)	9.0 (7.0–13.6)	0.754*
**In-hospital outcomes**						
Long LOS	127 (25.0)	25 (16.4)	0.054*	53 (25.6)	11 (22.0)	0.597
Rapid response team	2	2	0.214†	0	1	0.195†
House Officer calls	74 (14.6)	12 (8.3)	0.050	35 (16.9)	3 (6.0)	0.051
ICU admissions	9	2	0.1.000†	8	1	1.000†
ICU LOS, median (i.q.r.)	8.0 (3.0–15.0)	3.0 (3.0–3.0)	0.150*	4.0 (2.3–18.8)	n/a	n/a
Mortality	4	1	1.000†	1	0	1.000†
Reoperations	42	12	0.967	11	2	1.000†
**Unplanned diagnostics**						
CT	60 (11.8)	17 (11.8)	0.992	36 (17.4)	11 (22.0)	0.449
MRI	2	0	1.000†	3	0	1.000†
Complication rate	44	14	0.698	26	5	0.618
**Complication severity**	51	18	0.916†	32	5	0.790†
IIIa	9	4	0.730†	16	3	1.000†
IIIb	34	12	1.000†	7	1	1.000†
IVa	3	1	1.000†	5	1	1.000†
IVb	3	0	0.562†	3	0	1.000†
V	2	1	1.000†	1	0	1.000†
**Post-hospital outcomes**						
Readmissions	55 (10.8)	19 (13.2)	0.434	22	6	0.780
Readmissions, LOS, median (i.q.r.)	6.3 (4.0–9.4)	3.0 (2.0–7.5)	0.014‡*	6.5 (3.1–12.5)	5.5 (2.8–8.5)	0.397*
DAH_30_, median (i.q.r.)	25.0 (21.0–26.5)	25.5 (23.0–27.0)	0.001‡*	20.5 (14.0–24.0)	20.8 (16.4–23.0)	0.874*
**Discharge disposition**						
Independent	344 (67.9)	94 (65.7)	0.634	123 (59.4)	21 (42.0)	0.026‡
Home care	137 (27.0)	46 (32.2)	0.227	74 (35.7)	29 (58.0)	0.004‡
Rehabilitation centre	18	2	0.274	6	0	0.600†
Nursing home	2	0	1.000†	1	0	1.000†
Other ward	3	0	1.000†	2	0	1.000†
Deceased	2	1	0.526†	2	0	1.000†
Hospice	1	0	1.000†	0	0	n/a
**Frequency of post-hospital care**			0.223			0.635
1	113 (66.9)	26 (60.5)		39	18	
2	44	16		25	9	
≥ 3	12	1		9	2	
**Type of post-hospital care**						
ADL	65 (12.8)	19 (13.3)	0.883	51 (24.6)	14 (28.0)	0.624
Stoma	107 (21.1)	22 (15.4)	0.130	7	2	0.688†
Medication	33	15	0.108	44	11	0.908
Tube feeding	2	1	0.526†	15	5	0.566†
Wound care	25	13	0.061	32	6	0.536
Drain care	2	0	1.000†	12	12	0.000‡†
Catheter care	11	5	0.366†	2	1	0.479†
Other	3	0	1.000†	3	0	1.000†

Values are *n* (%) unless otherwise stated. ADL, activities of daily living; HPB, hepatopancreatobiliary; CMVS, continuous vital signs monitoring intervention; LOS, length of stay; DAH_30_, days alive at home 30 post-surgery. *Mann–Whitney U-test. †Fisher Exact test. ^‡^Statistically significant.

### Secondary outcomes

#### In-hospital outcomes

The number/percentage of nurse-to-HO calls was significantly lower in the intervention group, 7.5% *versus* 15.3% in the control group (*P* = 0.007; *Table 3*) and in the subgroup analysis for both groups (8.3% *versus* 14.6%; *P* = 0.05 in colorectal patients and 16.9% *versus* 6.0%; *P* = 0.051 in HPB patients; *Table 4*). None of the other outcomes differed statistically significantly between the CMVS and control groups including complication rates, the number of RRT calls and unplanned ICU admissions. This was also true for the post-hoc subgroup analysis (*Table 4*). Although overall complication rates did not differ between groups, a non-significant increase in complication severity IIIb (49.4% to 56.5%) and decrease in severity IVb (from 7.2% to 0%) was observed. A trend towards a reduced median ICU LOS in the CMVS group (3.0 *versus* 8.0 days) was observed.

#### Post-discharge outcomes

DAH_30_ was higher in the CMVS group (median 24.5 *versus* 24.0 days; *P* = 0.005) and more patients were discharged with a need for home care (38.9% *versus* 29.6%; *P* = 0.015). Although the readmission rate did not differ between groups, LOS of readmissions was lower in the control group (median 6.5 *versus* 4.0 days; *P* = 0.014). None of the other outcomes were different between the intervention and control groups (*Table 3*). In the post-hoc subgroup analysis, DAH_30_ and LOS of readmissions were different only in colorectal patients (*Table 4*). In HPB patients, more patients in the CMVS group were discharged with a need for care (59.4% *versus* 42.0%; *P* = 0.026), resulting in more patients who were discharged with a need for home care (58.0% *versus* 35.7%; *P* = 0.004).

#### Performed nursing activities

Based on trends assessments, 109 nursing activities were performed in 68 patients (35.1%) of which 70 (64.2%) were performed independently by the nurse and 39 (35.8%) in consultation with a physician (*Table 5*). Nurses independently performed nine (8.3%) additional measurements and 61 additional patient assessments resulting in wait-and-see (56.0%). In consultation with a physician, 10 (9.2%) diagnostic and 28 (25.7%) therapeutic interventions were performed.

**Table 5:** Performed nursing activities based on trend assessments

Nursing activity	*n* (%)
**Activity performed by nurse**	70 (100)
Patient assessment (wait-and-see)	61 (87.1)
Addition manual check measurement with MEWS	9 (12.9)
**Interventions performed in consultation with a physician**	39
Consulted physician but wait-and-see	1
Diagnostics	10
Blood test: blood culture	2
Chest X-ray	2
Electrocardiogram	1
CT scan	3
Blood test: arterial blood gas	1
Therapy	28
Analgesics	11
Oxygen supplementation	4
Bronchodilators	4
Fluid challenge	3
Beta-blockers	1
Diuretics	2
Breathing exercise	2
Digoxin	1

MEWS, Modified Early Warning Score; X-ray, energetic high-frequency electromagnetic radiation.

#### Patient experiences

A total of 163 questionnaires were completed (84%). Of patients, 76.7% (*n* = 125) rated the intervention 8 out of 10 or higher resulting in a median satisfaction of 8.0 out of 10 (i.q.r. 8–9; *Table 6*). Of patients, 83.4% (*n* = 136) found the intervention acceptable, resulting in a median (i.q.r.) acceptability of 4 out of 5 (3.6–5.0). The majority of patients found the patch easy to wear (88.6%), felt safer (71.2%) and would wear the patch again (92.6%). There were no significant differences between colorectal and HPB groups.

**Table 6:** Patient experience based on the questionnaire

	*n* = 163		
Acceptability score, range 0–5, median (i.q.r.)	4.0 (3.75–5.0)		
Satisfaction rating, range 0–10, median (i.q.r.)	8.0 (8.0–9.0)		
	Disagree (1–2)	Neutral (3)	Agree (4–5)
Comfort, range 0–5, median (i.q.r.)	6 (3.7)	11 (6.8)	145 (88.9)
Feeling safer, range 0–5, median (i.q.r.)	9 (5.5)	38 (23.3)	116 (71.2)
Wear again, range 0–5, median (i.q.r.)	2 (1.2)	10 (6.1)	151 (92.6)

In addition, patients made 47 remarks (*Table S3*). There were statements about the desire to have more insight into their own vital signs measurements and the results and impact of the CMVS intervention. Furthermore, most patients mentioned they were not bothered at all by wearing the sensor. In contrast, several patients mentioned negative aspects of the wearability of the sensor about it being too hard, especially when laying on their side in bed, and the need for replacement when diagnostic tests had to be done. Also, patients mentioned an increased feeling of safety by wearing the sensor.

## Discussion

In this study the effects of CMVS on the general ward on LOS and a broad range of other clinical outcomes in major abdominal surgery patients were explored. Adequate implementation of the CMVS intervention on the surgical ward was previously demonstrated and reported^34^. The results of the current study show that the addition of CMVS to the standard care was associated with a small, but statistically significant reduction in LOS. Besides, in the CMVS group, the number of nurse-to-HO calls was significantly reduced (15% to 8%). Based on trends assessments, 35% of patients received additional nursing activities. Patients highly accepted the CMVS intervention.

In the post-hoc subgroup analysis, the association of CMVS with reduced LOS was maintained in the colorectal group but not in the HPB group. This may be explained by a difference in the post-operative complication profile. Both surgery types may be complicated by anastomotic leaks, intra-abdominal abscess, or bleeding, all of which are accompanied by deviating vital signs^41^. In the HPB group, however, pancreatic resections result in delayed gastric emptying in 10–30% of patients, which delays normal oral intake and significantly prolongs LOS, but is not associated with deviating vital signs^42,43^.

Importantly, LOS in colorectal surgery has been significantly reduced since the introduction of Enhanced Recovery After Surgery (ERAS) protocols over a decade ago^44^. In this study the ERAS protocols were unchanged and strictly applied throughout the entire study period (including historical controls). The study period coincided in part with the coronavirus disease 2019 (COVID-19) pandemic, but this did not affect outcomes, because the care for elective major abdominal (mostly oncological) surgery patients was not affected in the hospital, as they were given priority over all other usual care.

Even though the observed reduction in LOS may suggest that CMVS has enabled more rapid detection and intervention in case of clinical deterioration, no significant differences were found in complication rate, complication severity, RRT calls, ICU admissions and ICU LOS. This study may not have had sufficient statistical power to determine differences in these rare outcomes. Nonetheless, a non-significant trend towards less-severe complications was noted in the CMVS group, which could have been the result of additional interventions triggered by early detection. Also, in the CMVS group we observed a trend towards a reduced median ICU LOS (3.0 *versus* 8.0 days), which is closely associated with the severity of complications^45^.

LOS is considered an important indicator for assessing the efficiency of hospital management, quality of patient care and functional evaluation^46^. From the point of view of the healthcare provider, a shorter LOS results in lower medical costs and increases bed capacity, which is especially important in times of scarcity as during the COVID-19 pandemic and ongoing nursing shortages^47–49^. However, early discharge may increase the need for home care and other resource utilization, which must be accounted for in total healthcare cost estimations^48^. This is supported by the finding showing increased use of home care in the CMVS group.

Given the successful implementation of CMVS in this study, nurses may have been more attentive to vital signs monitoring, resulting in proactive assessment of the patient condition allowing for accurate and thorough nursing care. In fact, 35% of patients received additional nursing activities based on trends assessments, including interventions such as optimizing analgesia, which may have contributed to timely patient discharge and reduced LOS. Also, fewer nurse-to-HO calls for both groups during evening and night shifts after implementation of CMVS were observed, which is important because it may reduce the burden on the on-call physicians^50,51^. Although this decrease is difficult to explain based on this study, it is possible that abnormalities were noticed earlier and were adequately dealt with by nurses, obviating the need for care escalation to physician on-call.

The present study used a proactive method of trend assessment every 4 h as opposed to a reactive method of threshold-based alarms for trend monitoring. In a previous feasibility study in the same hospital, an active alarm strategy impaired nurse acceptance and compliance, possibly due to alarm fatigue^25,28,29^. The optimal frequency of vital sign measurements on general wards is unknown, but should be high enough to detect early changes in vital signs well before the onset of life-threatening events^52^. Routine monitoring vital sign trends every 4 h without alarms may be considered adequate to detect vital sign trend deviations indicating an imminent systemic inflammatory response syndrome caused by postoperative complications^53–55^. More frequent monitoring assessments may not be needed, as vital signs monitoring in the general ward setting is not aimed at detecting severe acute events such as cardiac arrest.

Besides clinical outcome measures, other positive effects of CMVS on patient-centred outcomes are important to consider when assessing the utility of CMVS (or considering the pros and cons of implementing CMVS as standard care). For instance, this study shows that patient satisfaction and acceptance of the CMVS was very high. The perceived feeling of safety and high comfort of the sensor should be considered an important patient-reported outcome for the implementation of CMVS, especially considering these outcome measures scored much higher than other wearable devices in previous studies in which significant proportions prematurely discontinued the CMVS^56,57^. Comparison of results with prior studies is complicated given the heterogeneity in study designs, patient populations and outcomes and, more importantly, because of the wide range of different CMVS interventions, with regard to sensors, alarm strategy and follow-up of deviating vital signs.

In comparison with other literature, the results from previous studies on the impact of CMVS on LOS are diverse. In this study, the reduction in hospital LOS was modest but statistically significant. Two other before–after studies, in relatively large cohorts of medical and surgical patients with comparable LOS, did not show a significant reduction by CMVS^56,58^. Interestingly, one of these studies reported beneficial results for patients on unplanned ICU admissions and RRT calls^56^. One meta-analysis covering five studies showed a non-significant weighted mean reduction in LOS of 0.09 days^59^. One possible explanation for these results is that the incidence of major adverse events was rare, and therefore had no impact on median LOS. Another meta-analysis, covering three studies comparable to this study, did show a trend towards a reduction of LOS by a weighted mean reduction of 3.3 days^21^. However, confidence intervals were wide (−8.8 to 2.2 days) and therefore this meta-analysis failed to demonstrate a significant association between CMVS and LOS. A recent before–after study with a comparable intervention also showed a significant LOS reduction of 0.7 days^60^. However, the mean LOS in that study was twice as long as our colorectal group (8.0 days *versus* 4.0 days), which is not considered state-of-the-art when using ERAS protocols. Another study with a bed-based continuous monitoring device, measuring the same two vital signs, was consistent with the results of this study, showing a significant reduction in LOS of 0.4 days^58^. However, these patients could not be monitored during mobilization on the ward (only when supine).

Finally, the CMVS patch device used in this study was found to be highly acceptable to patients. This outcome was in line with previous studies using disposable finger probes or patches as devices^23,58^. In contrast, in another study, in 21% of patients a wrist-worn device was prematurely removed, indicating patient acceptability was relatively low compared to the patch device worn in our study^56^.

Important strengths of this study are the selective inclusion of highly complex abdominal surgery (colorectal and HPB), robust characterization of patient characteristics (including CCI and ASA scores) and an array of clinical outcome data, as well as the significant length of the study period. However, several limitations should be considered when interpreting the results. First, due to the before–after design, time trends and unobserved confounding factors may have affected changes in the outcomes and it precludes strong inferences regarding causal effects. An RCT therefore may be more suitable. On the other hand, the chosen design enabled assessment of the impact under ‘real-world conditions’, ensuring results may be better translatable to clinical practice than a RCT^30,61^. In fact, the complex nature of the CMVS intervention and implementation also impedes randomization of two different vital signs monitoring protocols in parallel on the same ward but could also lower the consent rate^62,63^. Furthermore, RCTs are costly and time-consuming, so not ideal for rapidly developing eHealth technologies such as CMVS systems^24,64^. Importantly, during the entire study period no changes were made in patient management or policies in, for example, EWS system and early discharge (ERAS) protocols. Although compliance with the ERAS items was not systematically measured, the hypothesis is that the ERAS items are routinely completed for all patients and that significant changes in compliance are unlikely. Additionally, no significant changes over time in median LOS data of historical controls during the study period were found. Furthermore, despite the COVID-19 pandemic, the clinical care and workflow for major abdominal surgery patients was unaffected and continued in a similar fashion in the hospital. Also, besides patient-, diagnosis- and intervention-related factors, LOS is determined by multiple factors unrelated to clinical outcomes such as discharge delay due to rehabilitation or home care capacity shortages^66–72^. Given the sizable control group and prolonged study period, it is assumed any variations in these factors were adequately controlled for in this study.

Furthermore, the study was limited to elective surgery and not emergency surgery. It is conceivable that the effects of the intervention are larger in emergency surgery patients given that postoperative complications occur more frequently in this group^65^. The study was conducted in a single hospital setting, and the results might not be generalizable to other institutions or types of hospitals.

There are also several statistical limitations. Given the exploratory nature of the study, many outcome measures were measured and compared. The multiple outcomes might have caused statistical multiplicity. For this reason, they should be interpreted as exploratory and *P*s are presented to three decimal places. In addition, the calculated sample size was not completely reached and this study may not have had sufficient statistical power to detect differences in rare outcomes (such as unplanned ICU admissions, RRT calls and ICU LOS). This is especially true for subgroup analysis.

Despite the promising findings, more robust prospective multicentre studies are needed to establish the true added value of CMVS for clinical care and analyse its causal effects on general wards. Such prospective trials should include a simultaneous evaluation of the quality and success of CMVS implementation, which is essential before any clinical value can be established. Analysis of the follow-up nursing activities on deviating trends and its consequences should also be included in such studies rather than just focusing on major patient outcomes such as complication severity, RRT calls, unplanned ICU admissions or mortality. All the proactive nursing activities collectively may contribute to the prevention of more serious complications and prolonged hospitalization times.

As an alternative to proactive trend assessment, machine-learning algorithms may be developed that provide reliable personalized clinical decision support tools to facilitate correct and timely interpretation of vital signs trends. This may contribute to the development of highly efficient alarm strategies. This will prevent unnecessary diagnostic procedures and overtreatment by reducing the number of irrelevant and false-positive alarms and may improve workflow efficiency on the ward^52,53,73,74^.

In addition, future availability of advanced and validated multiparameter CMVS wireless sensors, which are sufficiently accurate, patient-friendly and comprehensive, may allow the discontinuation of standard manual vital sign measurements by nurses, especially because measuring other vital signs (for example, body temperature and blood oxygen saturation) are still relevant for adequate detection of surgical complications. This may not only improve clinical outcomes to a greater extent^56^ but also reduce nursing workload and increase efficiency of inpatient care, which seems important for successful implementation of wearable CMVS systems on the ward^31^.

Considering that inpatient hospital stays are becoming increasingly shorter, postoperative complications and clinical deterioration will inevitably occur more frequently at home^75^. Therefore, continuing CMVS after discharge—which is possible with the sensor used in the study—may be considered to allow monitoring and timely detection at home^76^. This may further lower barriers for safe early discharge. In addition, functionality of providing patients with insight into their own vital signs *via* an app may generate more patient involvement in their own health and benefit recovery.

In conclusion, CMVS using wearable wireless sensors and proactive trend assessments was associated with a significant decrease in length of stay for colorectal surgery patients but not for HPB surgery patients. Although all other clinical outcomes were similar in both groups, a non-significant trend towards less-severe complications and reduced ICU LOS was noted in the CMVS group. CMVS with the sensor used in this study was highly accepted by patients. It is important to note that CMVS triggered additional nursing activities such as patient assessments and therapeutic interventions, which may eventually result in attenuation of the severity of postoperative complications. Future studies should focus on additional interventions prompted by CMVS and its consequences in carefully selected patient groups with a relatively high risk of deterioration to establish the causal effects of CMVS and enhance the quality and safety of postoperative care.

## Supplementary Material

zrad128_Supplementary_Data

## Data Availability

The data set of this study is available upon request by contacting the corresponding author.
