# Supplementary material for: Impact of wearable wireless continuous vital sign monitoring in abdominal surgical patients: before–after study
Source: BJS Open. 2024 Jan 17;8(1):zrad128. doi: 10.1093/bjsopen/zrad128 (PMC10794900; doi:10.1093/bjsopen/zrad128)
Supplement: zrad128_Supplementary_Data [file zrad128_supplementary_data.docx]

**Impact of wearable wireless continuous vital sign monitoring in abdominal surgical patients: a before-after study**

Jobbe PL Leenen^1,2,3^, Vera Ardesch^4^, Cor J Kalkman^5^, Lisette Schoonhoven^6,7^, Gijs A Patijn^1,2^

1. Department of Surgery, Isala, Zwolle, The Netherlands
2. Connected Care Centre, Isala, Zwolle, The Netherlands
3. Research Group IT Innovations in Healthcare, Windesheim University of Applied Sciences, Zwolle, The Netherlands
4. Flex pool department, Isala, Zwolle, The Netherlands
5. Department of Anaesthesiology, University Medical Centre Utrecht, Utrecht University, Utrecht, The Netherlands
6. Julius Centre for Health Sciences and Primary Care, University Medical Centre Utrecht, Utrecht University, Utrecht, The Netherlands;
7. School of Health Sciences, Faculty of Environmental and Life Sciences, University of Southampton, Southampton, UK.

**Corresponding author.**

Jobbe PL Leenen

Postal address: Dr. van Heesweg 2, 8025 AB, Zwolle

E-mail address: j.p.l.leenen@isala.nl

**ORCID ID**

0000-0001-7269-2892

**Supplementary Materials - Index**

|  |  |
| --- | --- |
| **Supplementary Appendixes** |  |
| Appendix S1 | *Page 2* |
| **Supplementary Figures and Tables** |  |
| Table S1 | *Page 3* |
| Table S2 | *Page 5* |
| Table S3 | *Page 7* |
| Figure S1 | *Page 8* |

**Supplementary Appendixes**

**Appendix S1: Modified Early Warning Score (MEWS) protocol**

The MEWS consist of the following elements; heartrate, systolic blood pressure (BP), respiratory rate, temperature, the level of consciousness is scored by the AVPU A = Alert V= Voice P= Pain U= Unresponsiveness (AVPU), saturation, urine production and a worry indicator. For all items cut off points are predetermined which will lead to an EWS score.

| **Score** | **3** | **2** | **1** | **0** | **1** | **2** | **3** |
| --- | --- | --- | --- | --- | --- | --- | --- |
| **MEWS** | | | | | | | |
| **Heartrate** |  | **<40** | **40-50** | **51-100** | **101-110** | **111-130** | **>130** |
| **Systolic BP** | **<70** | **70-80** | **81-100** | **101-200** |  | **>200** |  |
| **Respiratory rate** |  | **<9** |  | **9-14** | **15-20** | **21-30** | **>30** |
| **Temperature** |  | **<35,1** | **35,1-36,5** | **36,6-37,5** | **>37,5** |  |  |
| **AVPU** |  |  |  | **A** | **V** | **P** | **U** |
| **CMVS (D-EWS scores)** | | | | | | | |
| **Heartrate** |  | **<40** | **40-50** | **51-100** | **101-110** | **111-130** | **>130** |
| **Respiratory rate** |  | **<9** |  | **9-14** | **15-20** | **21-30** | **>30** |

- Saturation is not mandatory. It is only measured when needed according to agreements or own clinical judgement.
- If the patient uses oxygen the following parameter needs to be taken into account; saturation < 90% or > 90%. When <90%: add 3 points
- If urine production is < 75ml in past 4 hours: add 1 point
- If worried (nurses worry indicator) about patient condition: add 1 point

Response protocol to EWS score

| Score of 0-1: | Repeat EWS once a day |
| --- | --- |
| Score of 2: | Repeat EWS 3 times a day according EWS for the upcoming 24h (a 8 hours) (*monitoring frequency)* |
| Score > 3: | Contact physician, apply SBARR (communication model), within 30 minutes define treatment policy and evaluated status after 1 hour *(Clinical response)* |

**Supplementary Figures and Tables**

| **Table S1: Multivariate analysis of log transformed Length of Stay**  **Multivariate analysis of log transformed Length of Stay** | | | | | | | | | |
| --- | --- | --- | --- | --- | --- | --- | --- | --- | --- |
|  | **Total (n=908)** | | | **CR surgery (n=651)** | | | **HPB surgery (n=257)** | | |
|  | **Unstandardized coefficients (95% CI)** | **SD** | **p-value** | **Unstandardized coefficients (95% CI)** | **SD** | **p-value** | **Unstandardized coefficients (95% CI)** | **SD** | **p-value** |
| Constant | .322 (.238 - .406) | .043 | .004* | .422 (.330 - .514) | .047 | .000* | 1.062 (.868 – 1.257) | .099 | .000* |
| Gender | .007 (-.021 - .035) | .014 | .631 | .032 (.001 - .062) | .016 | .040* | -.016 (-067 - .036) | .026 | .551 |
| Surgery type | .141 (.105 - .177) | .018 | .000* | .091 (.055 - .126) | .018 | .000* | -.276 (-.338 - -.214) | .031 | .000* |
| Procedure | .202 (.166 - .238) | .019 | .000* | .177 (.131 - .223) | .024 | .000* | .125 (.070 - .180) | .028 | .000* |
| CCI score | .005 (.000 - .010) | .003 | .063 | .009 (.003 - .015) | .003 | .003* | .018 (.008 - .028) | .005 | .001* |
| Complications | .484 (.437 - .530) | .024 | .000* | .542 (.489 - .596) | .027 | .000* | .309 (.232 - .386) | .039 | .000* |
| Group | -.043 (-.077 - -.009) | .017 | .012* | -.058 (-.095 - -.021) | .019 | .002* | -.014 (-.078 - .050) | .033 | .675 |
| *statistically significant | | | | | | | | | |
| Abbreviations: CR: colorectal, HPB: hepato-pancreaticobiliary, n: frequency, CI: Confidence Interval, SD: standard deviation, CCI: Charlson Comorbidity Index | | | | | | | | | |

| **Multivariate analysis of log transformed Length of Stay with significant individual comorbidities** | | | | | | | | | |
| --- | --- | --- | --- | --- | --- | --- | --- | --- | --- |
|  | **Total (n=908)** | | | **CR surgery (n=651)** | | | **HPB surgery (n=257)** | | |
|  | **Unstandardized coefficients (95% CI)** | **SD** | **p-value** | **Unstandardized coefficients (95% CI)** | **SD** | **p-value** | **Unstandardized coefficients (95% CI)** | **SD** | **p-value** |
| Constant | .301 (.216 - .386) | .043 | .000* | .416 (.324 - .508) | .047 | .000* | 1.063 (.865 – 1.262) | .101 | .000* |
| Gender | .006 (-.021 - .034) | .014 | .647 | .031 (.001 - .062) | .016 | .044* | -.015 (-.067 - .036) | .026 | .560 |
| Surgery type | .152 (.115 - .189) | .019 | .000* | .093 (.057 - .128) | .018 | .000* | -.280 (-.345 - -215) | .033 | .000* |
| Procedure | .197 (.161 - .234) | .019 | .000* | .174 (.128 - .221) | .024 | .000* | .122 (.067 - .177) | .028 | .000* |
| CCI score | .001 (-.004 - .007) | .003 | .613 | .006 (-.001 - .013) | .004 | .085 | .019 (.008 - .029) | .005 | .001* |
| Complications | .485 (.439 - .532) | .024 | .000* | .544 (.490 - .598) | .027 | .000* | .313 (.236 - .390) | .039 | .000* |
| Group | -.033 (-.067 - -.002) | .018 | .041* | -.050 (-.088 - -.011) | .020 | .011* | -.011 (-.075 - .054) | .033 | .748 |
| Hypertension | .048 (.017 - .080) | .016 | .003* | .030 (-.006 - .067) | .019 | .105 | .013 (-.042 - .069) | .028 | .640 |
| COPD | -.010 (-.057 - .036) | .024 | .662 | -.006 (-.057 - .045) | .026 | .822* | -.079 (-.165 - .007) | .044 | .070 |
| *statistically significant | | | | | | | | | |
| Abbreviations: CR: colorectal, HPB: hepato-pancreaticobiliary, n: frequency, CI: Confidence Interval, SD: standard deviation, CCI: Charlson Comorbidity Index | | | | | | | | | |

| **Table S2: Analysis of over-time effect**  Table 1: median LOS, per year   \| **Year** \| **LOS, median (IQR)** \| \| --- \| --- \| \| 2019 (n=72) \| 5.5 (4.0-9.8) \| \| 2020 (n=357) \| 5.5 (4.0-9.5) \| \| 2021 (n=325) \| 5.5 (3.75-9.8) \| \| 2022 (n=154) \| - 1. (3.5-9.1) \|   Table 2: Results of the p-value of the Mann-Whitney U tests between years   \| **Comparison** \| **P-value of the Mann-Whitney U tests** \| \| --- \| --- \| \| Year 2019 vs. Year 2020 \| .800 \| \| Year 2019 vs. Year 2021 \| .865 \| \| Year 2019 vs. Year 2022 \| .111 \| \| Year 2020 vs. Year 2021 \| .899 \| \| Year 2020 vs. Year 2022 \| .041* \| \| Year 2021 vs. Year 2022 \| .036* \|   *significant with p<.05 |
| --- | --- | --- | --- | --- | --- | --- | --- | --- | --- | --- | --- | --- | --- | --- | --- | --- | --- | --- | --- | --- | --- | --- | --- | --- |

**Table S3: Patients remarks on the questionnaire (translated and adapted from Dutch)**

| **Desire to have more insight own vital signs (n=10)** |
| --- |
| 1. *I don't know how the intervention interacted with nurses and what it provides for them. However, a few days later it did show that signaling was done by nurses by the device. So definitely an asset for the future.* 2. *As a patient, I don't have any insight into the action or effect of this intervention. So I also have no opinion: neither positive nor negative.* 3. *I do understand the functionality and possible benefits of the intervention, but where have the patient insight in it?* 4. *I think it is important, that there is also communication from nurses to the patient about the monitoring . Now it felt like I wore the patch without purpose.* 5. *The most important function in my eyes is the intervention. However, this has not been necessary, is therefore difficult to assess for me.* 6. *It does not bother me, but also does not benefit me. I do not have insight in any results.* 7. *I think the sensor can help, but haven't actually noticed anything (probably because my vital signs were okay). (…)* 8. *I think it is important to provide feedback of the results to the patients. Otherwise it certainly does not match the patient's needs. On the last day upon discharge from the hospital, I was able to review my vital signs. Then you also understand the functionality of the system.* 9. *I would like to gain more insight in the relationship between interventions and the intervention. After application of the sensor, no feedback was provided to me.* 10. *No insight in the measurements and what the nurse assessed, it is difficult to assign a value judgment.* |
| **Comfort of the sensor (n=19)** |
| 1. *I did not notice anything.* 2. *The intervention did not give any objections.* 3. *(…). At least it wears comfortably.* 4. *The intervention didn't bother me.* 5. *I haven't noticed much, but think it's a great innovation.* 6. *The nice thing about the sensor is, you don't notice anything.* 7. *I have not been bothered in any way by the sensor.* 8. *The sensor is a too hard product. It was good to try once though.* 9. *It was fine, but I had to get used to it.* 10. *I did notice wearing it, super convenient!* 11. *The sensor is difficult to sleep with.* 12. *The sensor felt awkward on the stomach, but didn't notice anything else.* 13. *I don't feel anything of the senor.* 14. *I have not noticed much of the sensor.* 15. *Totally uncomfortable sensor, but actually felt safer with it.* 16. *It doesn't make me more happier, but it is useful.* 17. *The intervention is useful. I don't notice it much. It gives a kind of assurance that you are being watched.* 18. *Not bothered at all. (…)* 19. *I did not notice a lot of it (…)* |
| **Feeling more safe (n=5)** |
| 1. *It is a good thing there is proper control of patients.* 2. *I provided me an excellent reassured feeling.* 3. *In this case no negative development in the recovery process, so you don't notice anything. Still reassuring that the first signals are picked up.* 4. *(…) Actually felt safer with it.* 5. *(…) It gives a kind of assurance that you are being watched.* |
| **Benefits for healthcare professionals (n=4)** |
| 1. *I hope it will help in the future of health care.* 2. *I think the intervention can be of good service to nurses and patients.* 3. *It provides peace of mind for healthcare professionals.* 4. *It is important that healthcare professionals can monitor remotely.* |
| **Incompatible with diagnostics (n=2)** |
| 1. *The sensor had to be removed before going for a scan.* 2. *It was odd the sensor had to be removed before an electrocardiogram.* |
| **General positive comments (n=7)** |
| 1. I *always like new innovations.* 2. *It think it went well.* 3. *Great invention!* 4. *A very good initiative which provides new possibilities for the future.* 5. *Any initiative to improve care I think is fantastic!* 6. *Nice initiative to continue.* 7. *Research is always fine, a study is good for everyone.* |

**Figure S1: The Philips Healthdot wearable sensor**


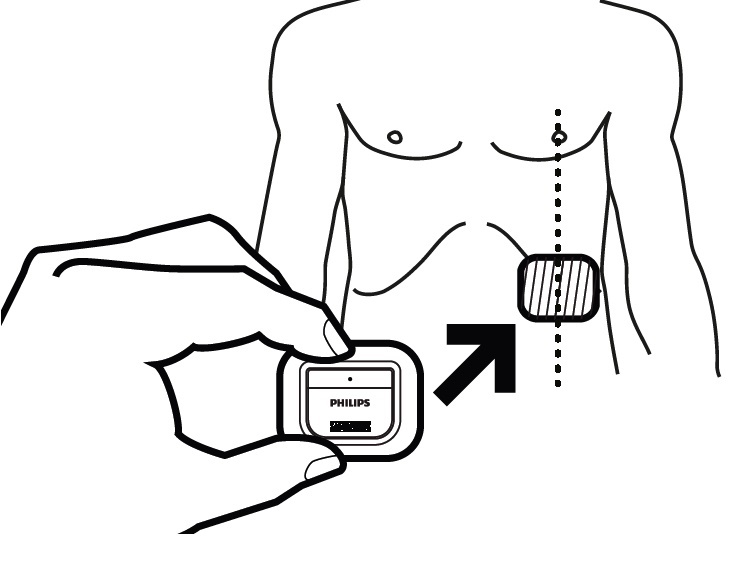


Reprinted from Philips Electronic Nederland BV under a CC BY license, with permission from Philips Electronic Nederland BV, original copyright 2020
